# Exploring Pharmacists’ Roles during the 2019–2020 Australian Black Summer Bushfires

**DOI:** 10.3390/pharmacy9030142

**Published:** 2021-08-19

**Authors:** Alexandra Moss, Toni Green, Simon Moss, Janique Waghorn, Mary-Jessimine Bushell

**Affiliations:** 1 Pharmacy Department, Faculty of Health, University of Canberra, Canberra, ACT 2617, Australia; u3172356@uni.canberra.edu.au (A.M.); toni.green@canberra.edu.au (T.G.); 2 College of Health and Human Sciences, Charles Darwin University, Casuarina, NT 0815, Australia; Simon.moss@cdu.edu.au; 3 Pharmacy Department, King’s College London, London WC2R 2LS, UK; janique.smit@kcl.ac.uk

**Keywords:** community pharmacy, pharmacist, care, natural disasters, bushfires, wildfires

## Abstract

Background: Australians are no strangers to sudden natural disasters, such as bushfires. The effects of a natural disaster can devastate local communities and health care services. Currently, limited research has explored the role of the pharmacist during a natural disaster. This study explores the role of the Australian pharmacist during the 2019/2020 Black Summer Bushfires. Methods: Semi-structured phone interviews were conducted with ten community pharmacists who worked through the Black Summer Bushfires whose daily tasks and work environment were directly affected by the bushfires. Thematic analysis using NVivo^®^, a qualitative data analysis software was conducted. Results: Analysis of the transcripts generated six main themes: collaboration; trauma and mental health; power and communication; acute presentations; triaging and emergency prescribing. Pharmacists worked in close collaboration with doctors and members of the local community. They provided triaging services, timely health advice about chronic health problems, and managed acute issues, including wound and burn management and mental health support in traumatic conditions, sometimes without power and communication amenities. The challenges presented to pharmacists during the bushfires warranted creative and flexible approaches at times. Conclusion: This study highlights the need for mental health support and training for pharmacists, provisional prescribing privileges, and a clearer set of contingency regulations and legislation related to emergencies and natural disasters. Further research is warranted to gain greater insight into the roles undertaken by Australian pharmacists during natural disasters and their autonomy in decision making processes during such times.

## 1. Introduction

Natural disasters have long been and remain a regular part of life for Australians. From September 2019 to February 2020, Australia was on fire. Bushfires across the country destroyed over 17 million hectares of land. Thirty-three people died (including nine firefighters), 3094 houses were destroyed, and an estimated 1 billion Australian animals were killed [1]. This bushfire season is now known as the ‘Black Summer’, and its effects were felt by pharmacists across the country, particularly along Australia’s east coast [2]. Commentators noted that, during the bushfires, pharmacists were transcending their roles. For example, some pharmacists were loading vehicles with supplies, including nappies, masks, asthma relievers, and other medicines and distributing them to local, temporary evacuation centres [2]. Staff employed in these pharmacies worked for weeks as fires roared towards their towns, without power and amidst thick smoke conditions [2].

In 2016, the International Pharmaceutical Federation published ‘Responding to disasters, guidelines for pharmacy’ [3]. The purpose of the document was to guide and enable pharmacy organisations across countries to prepare pharmacists for emergency situations, such as natural disasters. The document highlights the important role that pharmacists have during times of disaster, and guides pharmacists across the four broad phases of an emergency, prevention, preparation, response, and recovery.

To date, limited research, in the Australian context, has explored pharmacists’ role and preparedness in natural disasters such as bushfires [4,5]. This absence of evidence could create disparities between legislation and policy and the actual support and resources needed for pharmacists to ensure the continuous supply of medicines and professional services during challenging situations.

During disasters in Australia, communities expect pharmacies to remain open late outside of normal working hours. Sometimes pharmacists are asked to supply medicines without the consumer paying and outside of regulation (e.g., in lieu of a prescription) [6,7]. This presents challenging and sometimes ethically complex scenarios for pharmacists. For example, during the Tasmanian 2012/2013 bushfires, pharmacists were unable to support patients who sought Declared Schedule 4D (prescription only declared medicines e.g., benzodiazepines) and Schedule 8 (controlled medicines e.g., opioids) in the absence of their prescribing doctor [7]. This constraint was raised as a concern by pharmacists who indicated that regulations prevented palliative care patients from being able to access the Schedule 8 medicines they genuinely needed for pain relief [7,8]. The emergency supply regulation allows pharmacists to dispense a three-day supply of prescription medicines (excluding schedule 4D and schedule 8). Patients with chronic conditions that demand supplies beyond three days were referred to their nearest hospitals, even though road closures often precluded this alternative [7].

Australian emergency services and hospitals generally do not have the resources or capacity to accommodate individuals with chronic conditions that demand access to basic medical services rather than emergency care [7]. Consequently, during disasters, these patients struggle to access essential healthcare services and obtain a supply of their medicines. This impediment is the leading cause of death following disasters [9].

These chronic conditions encompass a broad range of ongoing and complex health conditions, including mental illness [10]. The prevalence of Australians living with a mental health condition, especially mental illnesses related to anxiety and depression, has escalated [11]. During a natural disaster, the likelihood that people may witness a trauma, and thus experience post-traumatic stress disorder (PTSD) surges [3,11]. Many patients will experience loss and grief having lost homes, pets, and sometimes family members or friends. Despite the impact of natural disasters on mental health, and the role pharmacists are expected to play during these times, there are currently no mandated crisis management or mental health first aid courses for pharmacy professionals in Australia.

Several studies have been conducted in Australia, the United States, Japan, and Canada, that highlight the role of the pharmacist during natural disasters. [12,13,14,15]. In comparison, published literature about the role of other health professionals’ during natural disasters, such as doctors and nurses, is plentiful.

Pharmacists’ roles have been expanded in some countries to prescribing and dispensing a 30-day emergency supply of medicines for patients with chronic conditions affected by natural disaster without a prescription [11,15]. One study showed that the majority (96.8%) of disaster and healthcare professionals surveyed believed pharmacists fulfil other roles in addition to dispensing and logistical supply management of medicines during natural disasters [16]. The two main barriers to greater participation were the ‘lack of understanding of what roles pharmacists can perform during a natural disaster’ and the ‘perceived turf encroachment or prejudices by other healthcare professionals’ [4,16]. Issues of role clarification and encroachment of other professionals could be overcome by clearly defining and recording the roles of pharmacists in a natural disaster.

Therefore, research is warranted to gain greater insight into Australian pharmacists’ roles during the black summer bushfires of 2019/2020. This research provides an opportunity to better understand how pharmacists work during a natural disaster and their critical roles and decision-making processes

## 2. Materials and Methods

A qualitative and retrospective thematic analysis design was chosen because limited research has explored the scope and range of Australian pharmacists’ roles during natural disasters. Data were collected via phone interviews until data saturation was achieved. Saturation was achieved after ten pharmacists were interviewed. Proceedings were audio-recorded and transcribed. Telephone interviews were chosen as the preferred method for conducting the study by the pharmacists because of the COVID-19 restrictions. In contrast to video conferences, this method enabled pharmacists to participate in a broader range of locations. Each interview was facilitated by the lead researcher with the aid of a list of semi-structured interview questions. The questions were developed by the research team and pre-tested and validated with registered practicing pharmacists prior to use. They were designed to enable pharmacists to share insights into their roles whilst reducing biases. The Consolidated criteria for reporting qualitative research (COREQ) was followed [17].

### 2.1. Sample Selection

As a result of the study being qualitative, the sample was purposive rather than random, and no power analyses were conducted [18,19]. Interviews were conducted until data saturation was reached, with no new influences or themes emerging. A total of ten pharmacists were interviewed.

### 2.2. Inclusion Criteria

Participants were registered community pharmacists working in a community pharmacy setting in the New South Wales South Coast region, whose daily tasks and work environment were directly affected by bushfires from December 2019 to February 2020.

### 2.3. Exclusion Criteria

To ensure pharmacists with previous years of work experience and training in the community pharmacy setting were included in the research intern pharmacists and pharmacists with less than 12 months’ work experience as a pharmacist were excluded.

### 2.4. Source of Participants

Participants were recruited and interviewed from September 2020 to November 2020. Participants were recruited using a mixture of purposeful and snowball sampling methods. Pharmacies located in the South Coast Region of New South Wales (east coast of Australia) were purposively contacted by telephone or email. Participants who fulfilled the inclusion criteria of the study were identified and invited to participate in the study. These participants were also granted the opportunity to identify and recruit other participants who also fulfilled the study criteria. Participants were deliberately recruited from diverse geographic locations across the NSW South Coast. The recruitment process remained open until data saturation was reached.

### 2.5. Obtaining Consent

In line with the social distancing requirements of COVID-19, this study was conducted as a paperless study. Eligible participants were provided with a digital copy of the Participant Information and Consent Form and asked to peruse and electronically sign.

### 2.6. Data Management and Analysis

Participants were screened to confirm eligibility and upon receipt of digital informed consent, a semi-structured interview was arranged and conducted over the telephone. The telephone was a COVID-19 safe, flexible, and cost-effective method for collecting data and allowed interviews to be conducted over a wider geographic area. It was a method most preferred by participants and accommodated their busy and conflicting work schedules. Limitations includ the inability to observe the behaviour and body language responses of participants and there were disruptions from work priorities as interviews were sometimes conducted whilst a pharmacist was at work. The lead researcher conducted all interviews, which lasted approximately 30 min each. The research team transcribed the data to facilitate the validation of the themes. Participants’ responses to the semi-structured interviews were digitally voice recorded by an audio device and recorded in field notes. These field notes were then transcribed verbatim into a Microsoft Word document. This document was secured in an electronic format and stored on password-protected computers at the University of Canberra. Only the lead researcher and project supervisors could access the raw data. The Word documents containing transcripts from the interview were uploaded to NVivo, a qualitative data management software program. Data were thematically coded manually by the lead researcher and project supervisors, enabling quality control of codifications. Using an inductive thematic analysis method, themes were identified and interpreted [20]. Each idea, concept or challenge emerging from the data was coded. Codes that correspond to the same underlying issues were converted to subthemes and themes. The data underwent several levels of analysis and coding to determine themes and sub-themes until a coherent story from the data evolved. The themes formed the basis of analytical interpretation.

### 2.7. Ethics Approval

This research project was approved by the Human Research Ethics Committee of the University of Canberra (HREC 4703).

## 3. Results

Analysis of the transcripts yielded six main themes:

- Collaboration
- Trauma and mental health
- Power and communication
- Acute presentations
- Triaging
- Emergency prescribing

*“A pharmacist’s traditional role in ensuring the continuous supply and safe use of medication for the community appeared to be their core role during the natural disaster. The challenges affecting this role brought upon by the bushfires warranted a creative approach at times, approaches that deviated from standard practices. Maintaining medication supplies for patients was impeded by the large influx of tourists into the region during the Christmas and New Year’s period of 2019/2020. Along with an increased population in the small communities were the challenges of road closures, displaced customers experiencing trauma, increased prevalence of respiratory conditions as well as power and communication service outages during this time, as a few pharmacists referred to as a “catastrophic disaster””*

(Pharmacists 2, 8, 10)

Collaboration with health professionals and the local community, communication and strong clinical knowledge were the types of roles that pharmacists used to overcome these challenges. The pharmacist’s role seemed to demand a balance between ensuring continuous and safe medication supply and addressing other issues such as acute presentations, mental health, emergency prescribing, and triaging.

### 3.1. Collaboration

#### 3.1.1. Doctors

Pharmacist participants highlighted the close working between themselves and general practitioners. Many saw this as the cornerstone for ensuring the continuous supply of medication, where supplies were available.

The relationship appeared to be synergetic. Doctors worked closely with emergency services to coordinate temporary evacuation centers and relied on pharmacists and their staff to supply medications for their doctor’s bags to support the hundreds of people sheltered at the center. Pharmacists also relied on general practitioners to provide prescriptions to ensure the continuous supply of medication to patients, especially for chronic conditions and staged or Schedule 8 Controlled Drugs.

*“Knowing the doctors and also having a good relationship with the doctors helped. Knowing that if you were to supply something, they would cover [the prescription] you for it.”*

Pharmacist 1

*“We [Pharmacists] provided emergency medications, Panadol, Nurofen and Dr’s Bag as per the doctors request and we were advised if anyone needed emergency medication at the time [at the evacuation center].”*

Pharmacist 8

#### 3.1.2. Local Community

Familiarity with the local community was repeatedly mentioned as an enabler for ensuring the continuous supply of medication during the bushfires and previous natural disasters. Due to the close, familiar, and accessible relationship between the pharmacists and their community, these individuals worked together during the bushfires. Pharmacists utilised every available resource for patients whose needs were more difficult to accommodate. To achieve this goal, they needed to work with local community members. For example, community members volunteered their boats to transport medication along the coast. Pharmacists also worked with the local community to identify vulnerable community members and deliver medicines to these people.

*“I had what [medications] was required and dispensed and one of the local, jet skiers, I guess you could call him, he was doing supply runs up and down the coast.”*

Pharmacist 3

#### 3.1.3. Other Pharmacies

The pharmacists interviewed described the value of communication and collaboration between pharmacists and other pharmacies in the region, enabling continuity of care and medication supply for patients. The ability to communicate with other pharmacies enabled swapping and transport of medication supplies; consequently, towns could access the necessary medication such as the short-acting beta-agonist salbutamol inhaler. This collaboration was mainly considered to be administrative.

*“We were able to call each other [organizational pharmacies], and work something out internally. So, we did have more advantage than one, independent, one-person pharmacies.”*

Pharmacist 2

#### 3.1.4. Pharmacy Staff

The close working relationships and teamwork between pharmacists and their pharmacy staff was fundamental to providing additional services. Many described, their own trauma of not knowing what was going to happen.

*“Our team at the pharmacy were incredible. I have never seen so much compassion, it was truly remarkable. So, I think it was we had an amazing team for we were supporting each other.”*

Pharmacist 7

#### 3.1.5. Isolation

In areas along the South Coast more heavily affected by fire damage, pharmacists experienced isolation and limited collaboration with others. Their isolation was exacerbated by loss of communication as a consequence of remoteness, road closures, and disruption to telephone lines, internet, electricity, and staff shortages because of family priorities. In most cases, pharmacists were not able to continue operating from their normal premises and contact with local doctors regarding prescribing was at times sporadic or non-existent. Some community members remained in these evacuated towns and pharmacists were instructed to remain operating by local authorities despite the catastrophic and unpredictable environment unfolding around them.

*“There was no relationship(s); I had no phone lines.”*

Pharmacist 6

#### 3.1.6. Hospital and Emergency Services

On a lesser scale, pharmacists contacted local hospitals and emergency services, but this contact appeared to mainly be facilitated by collaborating with general practitioners.

*“The hospital stayed open, the emergency department was really good and available to us, we could send people in that direction.”*

Pharmacist 7

### 3.2. Trauma and Mental Health

The theme of trauma was consistent throughout the study data and appears to be an important factor that determined the role of the pharmacists, shaping their working environment. Trauma was divided into three sub-themes: community, pharmacy, and medical. Trauma was experienced as stress, grief, and loss. This trauma was also experienced by some as medical emergencies and acute presentations. Pharmacists repeatedly commented on the trying and difficult working conditions during the bushfires.

*“It was very traumatic for all of us.”*

Pharmacist 7

The poor air quality at the time also contributed to distress in people with respiratory conditions, exacerbated by supply shortages of salbutamol inhalers. The loss of homes and personal belongings was common for pharmacy consumers and staff. The disorientation and distress felt by these people motivated pharmacists to provide mental health support and counselling.

People’s mental health was significantly impaired by the bushfire and support was provided by pharmacists across several different regions of the south coast during the bushfires, whether the pharmacists felt prepared to offer this support or not.

*“A lot of people were just managing to keep a lid on suppressing their anxiety and panic.”*

Pharmacist 2

*“The role of the pharmacist is to maximize the health of people by providing them with appropriate advice and medications that is right for their conditions and for their continuing health. As well as being somebody to talk to. A number of people were very distressed and wanted a shoulder to cry on.”*

Pharmacist 5

Most pharmacists felt they had not completed the training or preparation to manage natural disaster issues, in particular mental health. They had to depend on their existing experience to provide support. Many participants mentioned that their own traumatic experiences might have influenced their mental health support roles at the time.

### 3.3. Power and Communication

Pharmacists are heavily reliant on their ability to communicate with other individuals. Communication was identified as a strong enabler for pharmacists to undertake their roles during the bushfires. However, communication options for pharmacists depended on the path of the fires and their pharmacy locations. In some cases, mobile phone services or in-person communication were the only options available. No or limited communication was a common theme in all interviews. All pharmacists, to some degree, were affected by communication and power outages. Some pharmacies were more greatly affected than other pharmacies, compromising their ability to maintain normal roles and remain operating. Indeed, communication in an emergency was one theme that pharmacists have requested further training and assistance.

For many of the pharmacists, short message service (SMS) was the only available form of communication. An informal process of SMS telehealth was implemented. One pharmacist mentioned that the majority of local community members knew his mobile phone number. Pharmacists were also coordinating prescription and dispensing with doctors and the local community via SMS, including for Schedule 8 (controlled drug) medications.

*“For starters, we didn’t even have a telephone or mobile service… people are spread out, and within that area it’s heavily forested.”*

Pharmacist 3

*“I had to contact the doctors and the doctors contacted me via SMS with a prescription. We had no WiFi, no internet—the only thing working is SMS.”*

Pharmacist 10

Battery powered radio was at times the only information that pharmacists could access. Other participants communicated with the local community through Facebook and Messenger applications as well as by telephone to coordinate transport of medication supplies. In more dire circumstances, on premise signage were used to direct the local community to medical services, including the hospital.

*“We had to get as many torches as we could and gas lights just so we could see and dispense.”*

Pharmacist 6

Intermittent power outages, hot conditions, and road closures provided pharmacists with challenges. Without power, pharmacists were not able to access patient records or contact prescribers. Electronic payments were not available, culminating in shortages in the supply of medication. Some pharmacists mentioned that ordering additional stock in anticipation of a busy Christmas period prior to the bushfires helped to alleviate the burden of medication shortages as did medication swapping with other pharmacies.

*“We had no choice but to close. We couldn’t operate the pharmacy without power. It’s just not feasible.”*

Pharmacist 9

*“We didn’t have any access to patient records. We didn’t have access to difficult prescriptions.”*

Pharmacist 1

Participants mentioned that the regulation requiring medication to be dispensed from a registered pharmacy premise was an inconvenience during a bushfire. This inconvenience was especially pronounced for pharmacists working remotely with local doctors at the temporary evacuation centers.

*“At that time, I did call Medicare, to get some exemption but they would not budge. The challenge was we had to supply medication from the pharmacy site [for the evacuation centre].”*

Pharmacist 8

### 3.4. Acute Presentations

Pharmacists reported an increase in patients presenting with respiratory distress such as coughing and shortness of breath, chest pains, asthma, and emphysema exacerbations. One pharmacist had completed prior advanced training in administering oxygen supplementation. Due to the smoke and poor air quality, respiratory conditions were more prevalent and there was an increased demand for specific medicines. Stockpiling of preventive medication and short-acting beta-agonist relievers by customers was a concern for pharmacists. Use of spacers and patient education helped manage and reduce some patients expressed anxiety. However, other recommendations such as staying indoors to avoid the smoke appeared to be unachievable for some individuals.

*“My primary counselling point was always to encourage people to stay out of the smoke, but people would almost laugh at that.”*

Pharmacist 4

Patients also presented to the pharmacy with wounds and burns. One customer presented with severe burns that were three days old, even with a pharmacist referral, they refused to seek care from the hospital. The patient also appeared to be experiencing mental health issues at the time. Counselling and working with the patient’s family members were part of the patient care provided by the pharmacist.

### 3.5. Triaging

Pharmacists are mandated to operate from a registered premises, and so their roles were somewhat restricted to the confines of the pharmacy. Healthy, unwell, wounded, and vulnerable people all gathered at the local pharmacy. These locations were accessible healthcare services during the bushfires. Consequently, pharmacists undertook triaging from their premises. Information provided to the local community included addressing medication needs, managing people who were hurt and required acute medical care or support, providing directions to the hospitals, and information about which doctor was available.

*“We had someone outside triaging because people were panicking.”*

Pharmacist 7

*“Despite working in conditions with no electricity and communication issues, pharmacies tried to remain open but regarded their premises as a: ‘frontline on a battlefield.’”*

Pharmacist 10

### 3.6. Emergency Prescribing

Occasionally, pharmacists could not contact a doctor and needed to act timely and in the best interest of their patients and use their clinical knowledge to dispense medication without a prescription. Prednisolone liquid for an asthmatic child and insulin for adults with a history of prior use were supplied without a prescription. Some participants suggested that pharmacist prescribing abilities in communities without communication with a doctor would have been beneficial, particularly to manage respiratory conditions.

Some pharmacists expressed concerns about the balancing act between meeting patient needs and complying with regulations.

*“I think, as pharmacists, our concern is obviously following the law, and if you are ever unsure in your mind, it would be the sort of thing to err on the side of not wanting to do the wrong thing and denying supply. But you want to be helpful and there are a lot of pressures and you don’t want to be pressured into doing something that isn’t legal. When they [regulations] are changing on the fly during a crisis, it is much harder to adapt, then if we had say, emergency measures in place, that we were really familiar and comfortable with.”*

Pharmacist 4

## 4. Discussion

Pharmacists are recognized in Australia as the most accessible healthcare professional, particularly in rural and regional communities. This study repeatedly highlighted this accessibility during a natural disaster. During the bushfires, pharmacies were a place where healthy, unwell, wounded, and vulnerable people gathered. Pharmacists worked in close collaboration with doctors and members of the local community. They provided triaging services, and timely health advice about chronic health problems. They offered advice and care for acute issues such as wound and burns management. Pharmacists provided mental health support to people who had experienced trauma, loss, and grief, sometimes without power and communication amenities. The many challenges faced by pharmacists during the bushfires warranted a creative and flexible approach at times.

This study showed the need for pharmacists to triage and collaborate with other health professionals, provide mental health counselling, manage power and communication shortages, manage acute presentations of burns, eye, and respiratory problems and most importantly emergency prescribing, as necessary. Although pharmacists’ main role during the bushfires was to ensure the continuous supply and safe use of medication in the community, their roles extended beyond managing the coordination and dispensing of medication supplies. Our study has many of the themes of the Tasmanian bushfires study where pharmacists described working during the bushfires as difficult. Problems included loss of access to phones, power cuts, and stock loss due to issues with fridges. [21,22]. Pharmacists are highly trained health professionals, qualified to give advice on health issues and medicines and ensure the safe supply and use of medicines by the public. Medicines prevent, treat, or manage many illnesses or conditions and are the most common intervention in healthcare.

Collaboration was strong theme from the research findings, and there appeared to be a considerable overlap with the other emerging themes in the results. This highlights how pharmacists’ roles may be determined by their ability to collaborate with others. Power and geographical location facilitated communication, which in turn influenced a pharmacists’ level of collaboration. Those without power and communication were more likely to be isolated and relied more on their clinical decision making to provide patient-centred care, to dispense medications related to respiratory and endocrine conditions without a prescription. Their decision making was based on providing continuous patient-centred care, even at times outside regulation and policy. Those pharmacists who were more connected to the local community were able to provide triaging for patients requiring acute medical care and support and develop logistical relationships with other health professionals and the local community to ensure the continuity of care and medication supply to their patients.

Under emergency situations, pharmacists, particularly those working in isolation, used their clinical knowledge to dispense medicines without prescriptions in the best interest of their patients, and because of limited choice. Their decision making was based on providing continuous patient-centred care, even at times outside regulation and policy. In these circumstances, pharmacists often need to depend on their intuition, instead of regulations, yet this intuition can be impeded by the stress and anxiety that such conditions can evoke. The theme of emergency prescribing highlights the shift in decision making by pharmacists during the event of a nature disaster. How pharmacists make decisions is of increasing interest, sparked by a shift in the utilisation of the profession. This then calls into question whether the initial education and training of pharmacists is fit for purpose [23]. Literature suggests that pharmacists have been trained to work to prevent harm by correcting prescribers, rather than seeking to do good [24]. Where decision-making is motivated by the bioethical principle of non-maleficence (avoiding harm) rather than beneficence (doing good), decision-makers may be overly risk averse and more concerned with their role in any potential harm than working through the options that may be best for the patient. Early research has proposed a hierarchical model for decision-making consisting of four levels: submissive, corrective, consultive, and prescriptive [25]. In this work, pharmacists were found to mainly work at a submissive or corrective level, despite being competent to work at the higher levels. The findings presented in this paper builds upon this research, suggesting that, given the opportunity, pharmacists are able to rise to these higher levels of decision-making. However, this study also suggests that there needs to be an improvement in the way pharmacists are trained and supported, perhaps a shift to a more beneficence-focused decision-making training model.

Participants raised concerns about their ability to comply and remain familiar with the changing regulations and legislation during a natural disaster while maintaining ethical integrity and a duty of care to their patients. Governments and communities may need to consider a more explicit set of contingency regulations and legislation to manage emergencies before the occurrence of natural disasters. Pharmacists suggested that provisional prescribing privileges of medication-related to respiratory conditions may promote continuous care and improve health outcomes.

Another area where pharmacists stepped up and shifted towards a more beneficence focused role and decision-making was mental health. It is well known that bushfires have a significant effect on the health of a population especially respiratory and mental health conditions [26]. The prevalence and potential of mental health issues during and following a natural disaster is apparent. This study has highlighted that pharmacists play an important role in responding to the community’s mental health issues during a bushfire. Pharmacists are respected, well-positioned and easily accessible, particularly in regional and rural communities, to provide counselling and mental health support and advice. Pharmacists did, however, indicate they were not always equipped to provide these services during a natural disaster primarily because of limited training around these issues. They also spoke of their concerns for their own mental health and the mental health of other pharmacy staff in the aftermath of the bushfires. It has been noted that pharmacy staff are one of the highest risk groups for long-term psychological impacts [27]. The findings make it clear that the need for continued support for pharmacist to maintain resilience and good mental health—the recent COVID-19 pandemic has illustrated this even further, especially in Australia where those affected were still recovering from the Black Summer Bushfires [27].

Pharmacists in natural disaster-prone areas may benefit from mental health first aid training through continuing professional development. In addition, pharmacists will need to be aware of their scope of practice once a natural disaster such a as bushfire has occurred in their community. It has been shown that after a disaster prescription rates of mental health medications increase [28,29,30,31] and therefore pharmacy practice will need to be more aware of mental health counselling. In addition, pharmacists in bushfire prone areas may need to further professional education in burns, eye and respiratory problems [32,33]. Pharmacists in natural disaster-prone areas may benefit from mental health and counselling and support in the aftermath of a natural disaster such as a bushfire. Recently it has been found that pharmacists mental health during the pandemic has been affected [8]. So future support via policy and planning is needed by governments to assist the mental health workers who have survived the bushfires and other natural disasters.

Australia is vulnerable to natural disasters such as bushfires and the impact of bushfires can be devasting [21,34]. Learning from America and Canada has seen that preparedness for these natural disasters are a necessity [33,35]. For Australian universities and their pharmacy disciplines, curriculum must reflect the needs of pharmacists to be flexible and innovative during natural disasters and include the recommendations of this study of including high standard education of mental health counselling, focus on a doing good decision-making model as well as education on acute burns, eye, and respiratory problems. This current study shows the complexity of a pharmacist’s role during a natural disaster.

One limitation of this research is the questions revolved around the responses of individual pharmacists to the crisis and not the responses of employers, regulators, associations, government agencies, and other bodies. Consequently, the study explored the strategies and activities that promote individual resilience rather than system resilience [36], defined as the capacity of organisations, teams, communities, infrastructure, and other systems to withstand and to adapt in the aftermath of disruptive events [37]. Yet, the resilience of systems can significantly shape the resilience of individuals. To illustrate, when systems are vulnerable instead of resilient, individuals feel their roles and lives could shift unpredictably in the near future. The ensuing sense of instability tends to compromise resilience to change and other challenges [38,39]. Therefore, future studies could shift the sample and questions to explore the responses of employers, regulators, and other bodies.’

## 5. Conclusions

To facilitate a pharmacist’s complex and expanding role during a natural disaster, the study has highlighted the need to provide future mental health support and training for pharmacists, provisional prescribing privileges, a beneficence-focused decision-making education training model and a more straightforward set of contingency regulations and legislation related to emergencies and natural disasters. As the population continues to grow and inhabit more densely forested and remote regions of Australia, along with the increase in more severe weather patterns and experiences from the recent past, there is a heightened need for greater research in this area of pharmacy, pharmacists, and natural disasters.

## Data Availability

Data sharing not applicable.
